# Sight Impairment registration due to stroke—A small yet significant rise?

**DOI:** 10.1002/brb3.866

**Published:** 2017-10-29

**Authors:** Catey Bunce, Antra Zekite, Richard Wormald, Fiona Rowe

**Affiliations:** ^1^ Department of Primary Care & Public Health Sciences Kings College London London UK; ^2^ Institute of Ophthalmology, UCL London UK; ^3^ Department of Infectious and Tropical Diseases London School of Hygiene & Tropical Medicine London UK; ^4^ Research and Development Moorfields Eye Hospital NHS Foundation Trust London UK; ^5^ Department of Health Services Research University of Liverpool Liverpool UK

**Keywords:** blind, epidemiology, incidence, sight impairment, stroke

## Abstract

**Objectives:**

In the United Kingdom, when an individual's sight falls to and remains at a certain threshold, they may be offered registration as sight impaired. Recent analysis of causes of registrable sight impairment in England/Wales indicated that visual impairment due to stroke had increased as a proportionate cause of sight loss. We aim to assess whether there is evidence of an increase in incidence of certification for sight impairment due to stroke in England/Wales between 2008 and 2014.

**Materials and Methods:**

The number of certifications with a main cause of sight impairment being stroke was obtained from the Certifications Office London. Directly standardized rates per 100,000 were computed with 95% confidence intervals and examined. Poisson regression was used to assess evidence of trend over time.

**Results:**

In the year ending 31st March 2008, 992 people were newly certified with stroke with an estimated DSR of 2.1 (2.0 to 2.2) per 100,000 persons at risk. In the year ending March 31st 2014, there were 1310 certifications with a DSR of 2.5 (2.4 to 2.7). Figures were higher for men than women. Poisson regression indicated an estimated incidence rate ratio of 1.03 per year with 95% confidence intervals of 1.028 to 1.051, *P *< .001.

**Conclusions:**

These data suggest a small but statistically significant increase in the incidence of certifiable visual impairment due to stroke between 2008 and 2014. Figures are, however, considerably lower than estimated, perhaps suggesting that more should be done to address the visual needs of those who have suffered stroke.

## 1 INTRODUCTION

Visual field loss as a consequence to stroke is common. In particular, homonymous hemianopia is the most frequently reported type of visual field loss accounting for two‐thirds of visual field loss poststroke (Rowe, 2013). Visual field loss, inclusive of homonymous hemianopia, is reported to occur in up to 57% of stroke survivors in the acute stages of stroke (within 1 month of stroke onset) but falling in frequency to 8%–25% in the long‐term (Gilhotra, Mitchell, Healey, Cumming, & Currie, 2002; Gray et al., 1989; Hepworth et al., 2015; Zhang, Kedar, Lynn, Newman, & Biousse, 2006). This reduction in frequency relates to many factors including long‐term mortality, recovery of field loss and under reporting of field loss.

For those with persistent homonymous hemianopia there can be a considerable impact to quality of life and activities of daily living (Granger, Cotter, Hamilton, & Fiedler, 1993; Hepworth & Rowe, 2016; Jones & Shinton, 2006). If an individual's sight has reduced and remains below a particular level, their consultant ophthalmologist may offer registration as sight impaired (SI: partially sighted) or severely sight impaired (SSI: blind). Registration conveys certain benefits to the patient such as tax benefits and social support. The first step in the registration process is completion of a form known in England as the Certificate of Vision Impairment (CVI) and in Wales as the CVI‐W. Similar systems exist in Scotland and Northern Ireland. A copy of the CVI (CVI‐W) is sent to the patient's local social service department (or their agents) which triggers a needs assessment. Patients who have been registered visually impaired have reported real value—for some it may be a means to be put in touch with other people with similar conditions and share experiences, for others it is access to training or support for daily living (Boyce et al., 2014). One copy of the certificate is sent to the Certifications Office for epidemiological analysis. The Certifications Office is based at Moorfields Eye Hospital, London, United Kingdom, but operates under the auspices of the Royal College of Ophthalmologists.

An analysis of leading causes of certification for sight impairment in England and Wales had indicated that visual impairment due to stroke had increased as a proportionate cause of sight loss (Quartilho et al., 2016). It is quite possible that a proportionate decrease in one cause of sight loss (such as diabetes) might result in a proportionate increase in another (such as stroke). Such a change would not, however, impact on incidence. In this study, we wished to examine whether there was evidence of an increase in the incidence of certification for sight impairment due to stroke.

## 2 MATERIALS AND METHODS

When paper CVIs arrive at the Certifications Office, they are transcribed by trained coders and entered onto a database using a computer system which was developed and validated during a Guide Dogs funded project in 1998. A research assistant performs weekly validity checks and double data entry is conducted on a random sample of the data to ensure coding and consistency. Data captured include age at certification, gender, cause of certifiable visual loss and visual status—SSI, SI, or not stated. Diagnoses are captured for right and left eye separately and the ophthalmologist then selects the cause which in their view contributes most to sight impairment. In approximately 16% of certificates, the ophthalmologist is unable to determine a single cause, so multiple pathology is recorded. Because of this, when numbers due to any single cause are looked at, the proportion of certificates with a main cause and the proportion of certificates with a multiple cause, but the cause under investigation being contributory, are counted. This is the system adopted by Public Health England when reporting the public health indicator for sight loss in England. We obtained from the certifications office the numbers of individuals (by age and sex) for all certificates with:

1. a main cause of sight impairment being visual field defects (ICD9 code 368.4) or
2. stroke (cerebrovascular disease ICD9 codes 430‐438) (McCormick, Bhole, Lacaille, & Avina‐Zubieta, 2015; WHO, 1977) or
3. the main cause being determined as multiple, but a contributory cause being visual field defects or stroke,

for each financial year between 2008 and 2014, the dates being those for which the certifications office could provide data. Directly standardized rates were computed per 100,000 population in total and by gender and are presented with 95% confidence intervals computed by Byar's method (Breslow & Day, 1987). Poisson regression was used to assess the significance of the observed trend.

## 3 RESULTS

**Figure 1:**
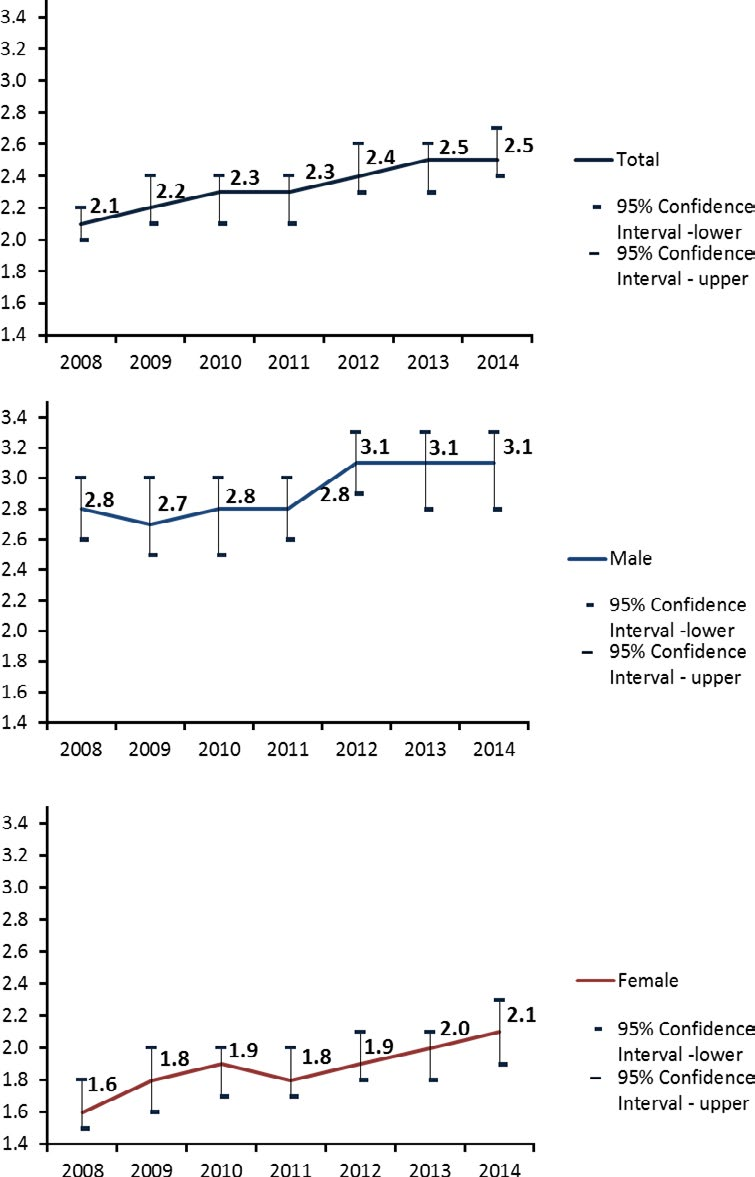
DSR of certifications with a main cause of vision loss being visual field defects or stroke or with a multiple cause but a contributory cause being visual field defects or stroke per 100,000 population in England and Wales in total and by sex

In the year ending 31st March 2008, 992 people were newly certified sight impaired due to stroke, with an estimated directly standardized rate of 2.1 (2.0 to 2.2) per 100,000 persons at risk (Table 1, Figure 1). Table 2 shows how many certificates in each year were attributed to different classifications and shows the total number of certificates completed for England and Wales in the corresponding financial year.

**Table 1:** Numbers of certifications due to stroke or visual field defects or due to a contributory cause being stroke or visual field defects

Year	Total	Male	Female	DSR Total	95% CI	DSR Male	95% CI	DSR Female	95% CI
2008	992	560	430	2.1	(2.0 to 2.2)	2.8	(2.6 to 3.0)	1.6	(1.5 to 1.8)
2009	1057	565	490	2.2	(2.1 to 2.4)	2.7	(2.5 to 3.0)	1.8	(1.6 to 2.0)
2010	1090	582	506	2.3	(2.1 to 2.4)	2.8	(2.5 to 3.0)	1.9	(1.7 to 2.0)
2011	1124	607	513	2.3	(2.1 to 2.4)	2.8	(2.6 to 3.0)	1.8	(1.7 to 2.0)
2012	1220	683	536	2.4	(2.3 to 2.6)	3.1	(2.9 to 3.3)	1.9	(1.8 to 2.1)
2013	1268	704	559	2.5	(2.3 to 2.6)	3.1	(2.8 to 3.3)	2.0	(1.8 to 2.1)
2014	1310	713	595	2.5	(2.4 to 2.7)	3.1	(2.8 to 3.3)	2.1	(1.9 to 2.3)

England and Wales in total and by sex and directly standardized rate (DSR) per 100,000 population in total and by sex with 95% confidence intervals (CI).

**Table 2:** Number of new certifications with a main cause of vision loss being visual field defects or stroke or with a multiple cause but a contributory cause being visual field defects or stroke: England and Wales

Year	Main cause stroke	Main cause visual field	Multiple cause stroke/visual field contributory	Total CVIS
2008	615	16	361	24057
2009	680	13	364	25498
2010	848	5	237	24233
2011	889	8	227	23926
2012	937	9	274	25079
2013	982	16	270	24009
2014	967	14	329	24213

There were more certificates for men than women (560 vs 430) and this was not as a result of age differences between the sexes since the DSR was statistically significantly higher in men than in women 2.8 (2.6, 3.0) vs 1.6 (1.5, 1.8). Over time there appears to have been a steady increase (albeit modest) in CVI rates due to visual field and stroke (Figure 1) until in the year March 31st 2014, the estimated directly standardized rate (95% confidence interval) was 2.5 (2.4 to 2.7)—statistically significantly higher than the figure observed in 2008. The total in 2008 was 992 registrations, rising consistently per annum to 1310 in 2014. Figures for men have remained consistently higher than in women (Table 1). The estimated incidence rate ratio per year was 1.03 with a 95% confidence interval of (1.028 to 1.051), *P* < .001.

The majority of certificates for stroke had a single main cause being stroke (615/992 in 2008 compared with 967/1310 in 2014). Figures for visual field defects alone were small. While the number of CVIs due to stroke has increased, Table 2 indicates that CVI figures overall have remained fairly constant over this time period.

## 4 DISCUSSION

These data suggest a small but progressive rise in the numbers of people certified as visually impaired due to visual field defects or stroke between 2008 and 2014. Wang, Rudd, and Wolfe (2013) examined survival rates of first‐in‐a‐lifetime strokes using the South London Stroke register between 1995 and 2011 and found that survival improved significantly over this time period. It is possible therefore that this increased survival from stokes is resulting in a higher incidence of certifiable sight impairment due to stroke although Douiri, Rudd, and Wolfe (2013) found little evidence of an increase in poststroke cognitive impairment.

It has been estimated that there are 100,000 new strokes per annum (The Stroke Association, 2016). There is a high percentage of visual field loss reported acutely in the stroke population (Hepworth et al., 2015). Furthermore, there is the potential that reported estimates of hemianopia are an underestimate where screening assessments are not sufficient or appropriate to detect visual field loss and, where stroke survivors do not complain of visual symptoms (Rowe, 2011, 2013). Taking these figures, conservative estimates of long‐term homonymous hemianopia due to stroke per annum range from 8000 to 25000 cases. Clearly, these numbers greatly exceed the average 1062 new CVI registrations due to stroke per annum. Numbers certified are likely to be lower than numbers sight impaired for a variety of reasons.

Barriers to certification have been evaluated (Boyce et al., 2014) and include uncertainty on when to certify, external pressures to reduce certification rates, perception of certification being the end of process rather than a route to services, poor awareness of benefits, incorrect assumptions about patients' views and lack of clarity regarding payment. Boyce and colleagues outline recommendations to address these barriers (2014). However, the barriers in detection of hemianopia and referral to vision services from stroke units must also be addressed. Detection of visual impairment on stroke units is reported as a potential problem with a lack of standardized assessment (Rowe, 2011). Stroke survivors do not always complain of visual field loss despite objective presence of hemianopia, either because they are not aware of it due to cognitive or visual inattention issues, or simply that they are not hampered by it in daily life (Rowe, 2013). Ten percent of stroke survivors with confirmed visual field loss were visually asymptomatic (Rowe, 2013). A lack of referral for formal visual evaluation is also common (Rowe, 2011, 2013). Even if the presence of hemianopia is suspected, certification cannot be undertaken if the patient is not referred to an ophthalmologist—the only professional authorized to complete the certificate.

The diagnosis of visual field loss is, however, important even for those who appear visually asymptomatic. It is important to raise awareness of the field loss and improve awareness to the affected side to help with detection of objects on that side and improve navigation (Jones & Shinton, 2006; Rowe, 2013). There can be considerable impact including altered mood, depression, impaired activities of daily living, increased falls, and reduced quality of life (Hepworth & Rowe, 2016).

There is a range of therapy options for homonymous hemianopia. A recent Cochrane systematic review (Pollock et al., 2011) states that there is benefit from therapy although the impact to functional outcomes remains to be determined. However, visual scanning exercises and other options are easily accessed in the NHS but must be provided by appropriately trained specialists (Pollock, Hazelton, & Brady, 2011; Rowe et al., 2015). It is important that these patients can access treatment in a systematic and appropriate manner. It is also important that they are offered CVI registration for persistent homonymous hemianopia.

The purpose of the CVI process is to provide a reliable route for individuals with sight impairment toward social care. Registration is provided by social services and is a voluntary process with a number of benefits such as additional help from local social services and potential eligibility for social sector benefits and tax concessions (if SSI). These benefits, along with provision of details of local social services and support organizations, can be outlined by Eye Clinic Liaison Officers (ECLOs) and Visual Rehabilitation Officers (VROs). It is recommended that part of the discharge pathway for these patients includes an appointment with the ECLO or signposting to local VRO services.

## 5 CONCLUSIONS

These data suggest a small but significant increase in the incidence of certifiable visual impairment due to stroke. The fact, however, that the numbers certified are so much lower that the projected numbers of new cases per annum (>8000) of homonymous hemianopia due to stroke suggests that significantly more needs to be done in order to ensure that all patients in need are certified. It has been argued that neglecting visual problems in patients with stroke can lead to increased incidence of injury and a deteriorating effect on rehabilitation and independence (Siong et al., 2014). It is therefore, important to improve detection of homonymous hemianopia, to provide timely advice and support to stroke survivors with homonymous hemianopia. It is equally important to ensure the CVI registration process is appropriately discussed with the patient and their carer, with access to support and with this process at repeated time points if necessary for those patients not initially ready to take on board this information. The role of the ECLO and VRO is recommended as a key part of this registration process.

## CONFLICT OF INTEREST AND DISCLOSURES

The data provided by Moorfields Eye Hospital, captured by the Certificate of Vision impairment (CVI) are Department of Health copyright and this work was made possible by collaboration with the Royal College of Ophthalmologists. Any views expressed in the publication are those of the author(s) alone and are not necessarily those of the Department of Health.
